# Social Media Use, Unhealthy Lifestyles, and the Risk of Miscarriage Among Pregnant Women During the COVID-19 Pandemic: Prospective Observational Study

**DOI:** 10.2196/25241

**Published:** 2021-01-05

**Authors:** Xiaotong Zhang, Jue Liu, Na Han, Jing Yin

**Affiliations:** 1 School of Medicine Macau University of Science and Technology Macau China; 2 Department of Epidemiology and Biostatistics School of Public Health Peking University Beijing China; 3 Department of Obstetrics and Gynecology Maternal and Child Health Hospital of Tongzhou District Beijing China

**Keywords:** COVID-19, social media use, miscarriage, cohort study, pregnancy, pregnant women, social media, China, risk, prospective, online health information

## Abstract

**Background:**

The COVID-19 pandemic has resulted in changes to normal life and disrupted social and economic function worldwide. However, little is known about the impact of social media use, unhealthy lifestyles, and the risk of miscarriage among pregnant women during the COVID-19 pandemic.

**Objective:**

This study aims to assess the association between social media use, unhealthy lifestyles, and the risk of miscarriage among pregnant women in the early stage of the COVID-19 pandemic in China.

**Methods:**

In this prospective cohort study, 456 singleton pregnant women in mainland China were recruited during January and February 2020. Sociodemographic characteristics, history of previous health, social media use, and current lifestyles were collected at baseline, and we followed up about the occurrence of miscarriage. Log-binomial regression models were used to estimate the risk ratios (RRs) of miscarriage for women with different exposures to COVID-19–specific information.

**Results:**

Among all the 456 pregnant women, there were 82 (18.0%) who did no physical activities, 82 (18.0%) with inadequate dietary diversity, 174 (38.2%) with poor sleep quality, and 54 (11.8%) spending >3 hours on reading COVID-19 news per day. Women with excessive media use (>3 hours) were more likely to be previously pregnant (*P*=.03), have no physical activity (*P*=.003)*,* have inadequate dietary diversity (*P*=.03), and have poor sleep quality (*P*<.001). The prevalence of miscarriage was 16.0% (n=73; 95% CI 12.6%-19.4%). Compared with women who spent 0.5-2 hours (25/247, 10.1%) on reading COVID-19 news per day, miscarriage prevalence in women who spent <0.5 hours (5/23, 21.7%), 2-3 hours (26/132, 19.7%), and >3 hours (17/54, 31.5%) was higher (*P*<.001). Miscarriage prevalence was also higher in pregnant women with poor sleep quality (39/174, 22.4% vs 34/282, 12.1%; *P*=.003) and a high education level (66/368, 17.9% vs 7/88, 8.0%; *P*=.02). In the multivariable model, poor sleep quality (adjusted RR 2.06, 95% CI 1.24-3.44; *P*=.006), 2-3 hours of media use daily (adjusted RR 1.74, 95% CI 1.02-2.97; *P*=.04), and >3 hours of media use daily (adjusted RR 2.56, 95% CI 1.43-4.59; *P*=.002) were associated with miscarriage. In the sensitivity analysis, results were still stable.

**Conclusions:**

Pregnant women with excessive media use were more likely to have no physical activity, inadequate dietary diversity, and poor sleep quality. Excessive media use and poor sleep quality were associated with a higher risk of miscarriage. Our findings highlight the importance of healthy lifestyles during the COVID-19 pandemic.

## Introduction

The COVID-19 pandemic, caused by the novel coronavirus SARS-CoV-2, has resulted in changes to normal life and has disrupted social and economic functions worldwide. It was reported that there were 52,487,476 confirmed cases and 1,290,653 deaths as of November 13, 2020 [1]. Compared with seasonal influenza, COVID-19 has a higher case-fatality ratio (0.98%-5.9% vs 0.1%) and infectivity (R_0_: 2.3-6.2 vs 1.2-1.4) [2-5]. Mortality in patients with COVID-19 has been associated with age and comorbidities (eg, hypertension, diabetes, and cardiovascular diseases) and differed across countries [6,7]. To date, no specific treatment has been found for COVID-19 and supportive measures have been used for patients with COVID-19. Nonpharmacologic interventions remain the key for curbing the spread of the virus, including active case finding and management, identification and quarantine of close contacts, social distancing, and personal protection (eg, hand hygiene and face mask use) [5]. China has taken strict measures to prevention and control of the pandemic, especially on social distancing and social isolation during the early stage of the pandemic. Wuhan City suspended all transportation in and out of the city from January 23 to April 8, 2020. Intra-area and interarea transportation restrictions were applied throughout the entire country of China, from big cities to small villages, from January to February 2020.

Along with the transmission of SARS-CoV-2, the information related with COVID-19 was also spread rapidly. One of the most accessible and fastest platforms for broadcasting information is social media. It represents a conglomerate of electronic platforms used for creating and sharing information, ideas, messages, etc [8]. Social media has become the major source of information about COVID-19. It enabled rapid and widespread reach of public health communications to help individuals take timely self-protection interventions. However, the speedy spread of COVID-19 worldwide also became a source of public worry, and several unknowns regarding this new pathogen created a state of panic [9]. Previous studies have shown that media coverage of COVID-19–related news induced fear and caused psychological stress during geographical lockdowns, extended quarantines, and financial and social hardships [9].

Miscarriage is a common adverse pregnancy outcome and one of the major public health problems. Miscarriage refers to a spontaneous demise of pregnancy before the fetus reaches viability [10]. Approximately 25% of pregnancies end in miscarriage, most occurring within early pregnancy (<13 weeks) [11]. Although the causes of miscarriage have not been fully explained, previous studies have shown a negative association of advanced maternal age (≥35 years), tobacco use, psychological problems, BMI, and other unhealthy lifestyles with miscarriage [12-14]. Excessive media consumption about COVID-19 was reported to be associated with increased anxiety in the general population in a cross-sectional study conducted in Russia [15]. Currently, little is known about the association between social media use, unhealthy lifestyles, and the risk of miscarriage among pregnant women. In this prospective cohort study, we aim to assess the association between social media use, unhealthy lifestyles, and the risk of miscarriage among pregnant women in the COVID-19 pandemic.

## Methods

### Study Design

This was a prospective cohort study conducted in a tertiary maternal and child health hospital in Beijing, China. The hospital was responsible for the prenatal care of all pregnant women living in Tongzhou district of Beijing. The primary aim of this cohort study is to investigate the short- and long-term health effects of prenatal exposures (eg, poor sleep quality) on mothers and their children. Baseline recruitment was conducted in January and February 2020, and pregnant women who visited the outpatient clinic for the first prenatal examination at Tongzhou Maternal and Child Health Hospital were recruited when they met the following inclusion criteria: <14 gestational weeks, singleton pregnancy, plan to have antenatal care and delivery in Tongzhou Maternal and Child Health Hospital, and resided in Tongzhou during the past half year and have no plan to move out after delivery. The study was approved by the institutional review boards at Peking University (IRB00001052–18003), and all participants gave written informed consent at the enrollment.

Baseline information was collected at the first prenatal visit by trained medical workers through a standardized questionnaire, such as sociodemographic characteristics (age, educational level, region, and family income), history of previous health (cesarean section, preterm birth, miscarriage, and fist pregnancy or not), prepregnancy weight, smoking status, physical activities, dietary diversity, and sleep quality. There were 504 pregnant women that met the inclusion criteria and were recruited at baseline. By August 2020, 11 women moved out of Tongzhou, 27 women were lost to follow-up, and 10 women transferred to other hospitals. Finally, the remaining 456 participants were included in this study.

### Assessment of Media Use About COVID-19

We collected the information on media use about COVID-19 by the following question: “How long did you spent on reading COVID-19 news every day from social media (official or unofficial)?” Participants were divided into five COVID-19–specific information exposure groups (<0.5 hours, 0.5 hours-1 hour, 1 hour-2 hours, 2-3 hours, and >3 hours) by their answers regarding the time spent on reading COVID-19 news. Because of the similar prevalence of miscarriage in the 0.5 hours-1 hour and 1 hour-2 hours groups in this study, we combined the two groups into one (0.5-2 hours) group as the reference group in the final analysis.

### Follow-up for Pregnancy Outcomes

The follow-up of the pregnancy outcomes was conducted by local medical workers. Pregnant women took regular antenatal care and delivered in the hospital. The information on pregnancy outcomes was obtained though the medical electronic information system in the hospital, which automatically recorded information during each antenatal care and delivery. Miscarriage is defined as a pregnancy loss that occurs before 20 completed weeks of gestational age [10]. The prevalence of miscarriage was defined as the proportion of participants who had a miscarriage to all participants.

### Covariates

Covariates were collected at the first prenatal visit, including age, educational level, region, family income, history of cesarean section, history of preterm birth, history of miscarriage, gravidity, prepregnancy weight, smoking status, physical activities, dietary diversity, and sleep quality. Prepregnancy BMI was calculated using weight (in kilograms) divided by the square of height (in meters). We assessed dietary diversity using nine food groups, as reported in previous studies [16]. The participants reported their consumption frequencies of various food groups, including meat, vegetables, fish, eggs, fruits, legumes, milk, rice, and nuts. The dietary diversity score (DDS) was calculated, with scores ranging from 0 to 9. Inadequate dietary diversity was defined as DDS<7 [16]. Sleep quality was evaluated by the Pittsburgh Sleep Quality Index (PSQI) [17]. The PSQI is the gold standard questionnaire for assessing subjective sleep quality and is framed in a 4-point Likert scale (0-3) analyzing seven factors, including subjective sleep quality, sleep duration, sleep latency, habitual sleep efficiency, sleep disturbances, use of sleeping medications, and daytime dysfunction [17]. The scores from each component were added to give a sum score, also called a total score (range 0-21). Poor sleep quality was defined as a PSQI≥5 in the pregnant women [18].

### Statistical Analysis

Mean (SD) values and proportions of baseline characteristics were calculated. We calculated the mean (SD) for age. We used proportions to describe baseline characteristics of pregnant women, such as age group, region, and educational levels, and the chi-square test or Fisher exact probability test was used to compare the distributions of baseline characteristics according to time spent on reading COVID-19 news. Prevalence of miscarriage and its 95% CI was calculated. Miscarriage prevalence in women with different characteristics were also compared using chi-square test or Fisher exact probability test.

Multivariable log-binomial regression models were used to estimate the adjusted risk ratios (RRs) and their 95% CIs of miscarriage for women with different exposures of COVID-19–specific information. Women were divided into four exposure groups (<0.5 hours, 0.5-2 hours, 2-3 hours, and >3 hours) by their answers regarding the time spent on reading COVID-19 news. Women who spent 0.5-2 hours on reading COVID-19 news per day were set as the reference group in the final analysis. In the multivariable model, we additionally adjusted for other potential risk factors, including age group (<35 years or ≥35 years), educational level (high school or below, or college or above), region (rural or urban), family income (<¥5000 [US $764], ¥5000-¥10,000 [US $764-$1528], or >¥10,000 [US $1528]), history of cesarean section (no or yes), history of preterm birth (no or yes), history of miscarriage (no or yes), first pregnancy (no or yes), prepregnancy BMI (underweight, normal weight, overweight, or obese), smoking (nonsmoker, previous smoker, or current smoker), physical activities (never, sometimes, usually, or every day), inadequate dietary diversity (no or yes), and poor sleep quality (no or yes) by backward methods. To examine the robustness of our findings, we did sensitivity analyses by adjusted covariates in the multivariable models as continuous variables for several variables (age and DDS), instead of categorical variables. In the subgroup analysis, we divided women into different subgroups by baseline characteristics (region, age group, and history of miscarriage). Among these baseline subgroups, we examined the associations between time spent on reading COVID-19 news and the risk of miscarriage after adjusting for other potential risk factors. All the analyses were done with SAS software, version 9.4 (SAS Institute). Two-sided *P* values less than .05 were regarded as statistically significant.

## Results

### Baseline Characteristics

Among all the 456 pregnant women included, 84.2% (n=384) were younger than 35 years, 54.4% (n=248) were living in an urban area, 9.2% (n=42) had a history of miscarriage, 56.6% (n=258) were having their first pregnancy, 28.1% (n=128) were overweight or obese, 7.9% (n=36) were a previous or current smoker, 18.0% (n=82) did no physical activity, 18.0% (n=82) had inadequate dietary diversity, and 38.2% (n=174) had poor sleep quality. The mean age at baseline was 30.0 (SD 4.2) years.

### Time Spent on Reading COVID-19 News and Unhealthy Lifestyles

The mean time spent on reading COVID-19 news was 1.8 (SD 0.9) hours per day. Of the 456 pregnant women, only 23 (5.0%) spent less than 0.5 hours on reading COVID-19 news per day, whereas 247 (54.2%) women spent 0.5-2 hours and 54 (11.8%) women spent >3 hours on reading COVID-19 news per day. Women with excessive media use (>3 hours) were more likely to be previously pregnant (*P*=.03), have no physical activity (*P*=.003), have inadequate dietary diversity (*P*=.03), and have poor sleep quality (*P*<.001; see Table 1).

**Table 1:** Baseline characteristics of pregnant women by time spent on reading COVID-19 news.

Characteristics		Total (N=456)	Time spent on reading COVID-19 news (hours), n (%)				*P* value
			<0.5 (n=23)	0.5-2 (n=247)	2-3 (n=132)	>3 (n=54)	
**Age group (years)**							.65
	<35	384 (84.2)	19 (82.6)	211 (85.4)	107 (81.1)	47 (87.0)	
	≥35	72 (15.8)	4 (17.4)	36 (14.6)	25 (18.9)	7 (13.0)	
**Educational level**							.29
	High school or below	88 (19.3)	40 (16.2)	5 (21.7)	29 (22.0)	14 (25.9)	
	College or above	368 (80.7)	207 (83.8)	18 (78.3)	103 (78.0)	40 (74.1)	
**Region**							.48
	Rural	208 (45.6)	7 (30.4)	117 (47.4)	59 (44.7)	25 (46.3)	
	Urban	248 (54.4)	16 (69.6)	130 (52.6)	73 (55.3)	29 (53.7)	
**Family income, ¥5000-¥10,000 (US $764-$1528)**							.11
	<5000	76 (16.7)	2 (8.7)	47 (19.0)	17 (12.9)	10 (18.5)	
	5000-10,000	222 (48.7)	14 (60.9)	105 (42.5)	72 (54.5)	31 (57.4)	
	>10,000	158 (34.6)	7 (30.4)	95 (38.5)	43 (32.6)	13 (24.1)	
**History of cesarean section**							.71
	No	394 (86.4)	18 (78.3)	215 (87.0)	114 (86.4)	47 (87.0)	
	Yes	62 (13.6)	5 (21.7)	32 (13.0)	18 (13.6)	7 (13.0)	
**History of preterm birth**							.09
	No	444 (97.4)	23 (100.0)	243 (98.4)	128 (97.0)	50 (92.6)	
	Yes	12 (2.6)	0 (0.0)	4 (1.6)	4 (3.0)	4 (7.4)	
**History of miscarriage**							.32
	No	414 (90.8)	19 (82.6)	229 (92.7)	118 (89.4)	48 (88.9)	
	Yes	42 (9.2)	4 (17.4)	18 (7.3)	14 (10.6)	6 (11.1)	
**First pregnancy**							*.03^a^*
	No	198 (43.4)	8 (34.8)	99 (40.1)	58 (43.9)	33 (61.1)	
	Yes	258 (56.6)	15 (65.2)	148 (59.9)	74 (56.1)	21 (38.9)	
**Prepregnancy BMI**							.62
	Underweight	58 (12.7)	4 (17.4)	34 (13.8)	13 (9.8)	7 (13.0)	
	Normal weight	270 (59.2)	11 (47.8)	153 (61.9)	75 (56.8)	31 (57.4)	
	Overweight	82 (18.0)	6 (26.1)	36 (14.6)	28 (21.2)	12 (22.2)	
	Obese	46 (10.1)	2 (8.7)	24 (9.7)	16 (12.1)	4 (7.4)	
**Smoking**							.48
	Nonsmoker	420 (92.1)	23 (100.0)	226 (91.5)	121 (91.7)	50 (92.6)	
	Previous smoker	32 (7.0)	0 (0.0)	17 (6.9)	11 (8.3)	4 (7.4)	
	Current smoker	4 (0.9)	0 (0.0)	4 (1.6)	0 (0.0)	0 (0.0)	
**Physical activities**							*.003*
	Never	82 (18.0)	0 (0.0)	39 (15.8)	24 (18.2)	19 (35.2)	
	Sometimes	230 (50.4)	10 (43.5)	124 (50.2)	73 (55.3)	23 (42.6)	
	Usually	124 (27.2)	10 (43.5)	73 (29.6)	31 (23.5)	10 (18.5)	
	Every day	20 (4.4)	3 (13.0)	11 (4.5)	4 (3.0)	2 (3.7)	
**Inadequate dietary diversity (DDS^b^ score<7)**							*.03*
	No	374 (82.0)	21 (91.3)	209 (84.6)	107 (81.1)	37 (68.5)	
	Yes	82 (18.0)	2 (8.7)	38 (15.4)	25 (18.9)	17 (31.5)	
**Poor sleep quality (PSQI^c^ score≥5)**							*<.001*
	No	282 (61.8)	21 (91.3)	193 (78.1)	56 (42.4)	12 (22.2)	
	Yes	174 (38.2)	2 (8.7)	54 (21.9)	76 (57.6)	42 (77.8)	

^a^Italics indicate significant *P* values.

^b^DDS: dietary diversity score.

^c^PSQI: Pittsburgh Sleep Quality Index.

### Prevalence of Miscarriage by Time Spent on Reading COVID-19 News and Unhealthy Lifestyles

**Figure 1:**
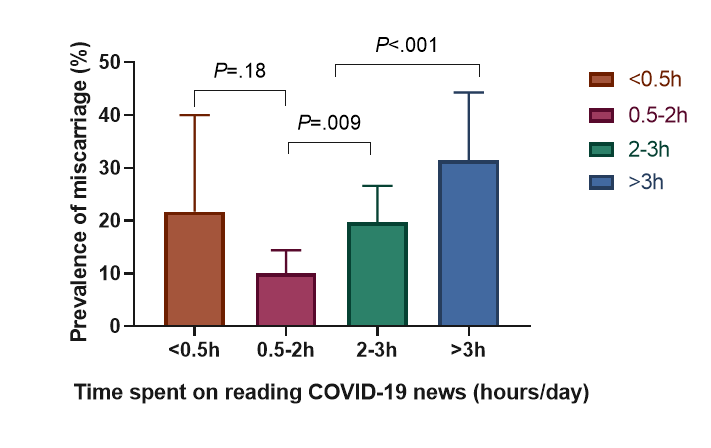
Comparison of miscarriage prevalence in pregnant women by time spent on reading COVID-19 news.

Out of 456 pregnant women, 73 had a miscarriage. The prevalence of miscarriage was 16.0% (95% CI 12.6%-19.4%). Compared with women who spent 0.5-2 hours (25/247, 10.1%) on reading COVID-19 news per day, miscarriage prevalence in women who spent <0.5 hours (5/23, 21.7%), 2-3 hours (26/132, 19.7%), and >3 hours (17/54, 31.5%) were higher (*P*<.001; see Figure 1). Miscarriage prevalence was also higher in pregnant women with poor sleep quality (39/174, 22.4% vs 34/282, 12.1%; *P*=.003) and a high education level (66/368, 17.9% vs 7/88, 8.0%; *P*=.02; see Table 2).

**Table 2:** Prevalence of miscarriage in pregnant women by different baseline characteristics.

Characteristics		Participants, n	Miscarriage prevalence, n (%)	*P* value
Total		456	73 (16.0)	N/A^a^
**Age group (years)**				.85
	<35	384	62 (16.1)	
	≥35	72	11 (15.3)	
**Educational level**				.02
	High school or below	88	7 (8.0)	
	College or above	368	66 (17.9)	
**Region**				.17
	Rural	208	28 (13.5)	
	Urban	248	45 (18.1)	
**Family income, ¥5000-¥10,000 (US $764-$1528)**				.06
	<5000	76	7 (9.2)	
	5000-10,000	222	44 (19.8)	
	>10,000	158	22 (13.9)	
**History of cesarean section**				.73
	No	394	64 (16.2)	
	Yes	62	9 (14.5)	
**History of preterm birth**				.39
	No	444	70 (15.8)	
	Yes	12	3 (25.0)	
**History of miscarriage**				.57
	No	414	65 (15.7)	
	Yes	42	8 (19.0)	
**First pregnancy**				.14
	No	198	26 (13.1)	
	Yes	258	47 (18.2)	
**Prepregnancy BMI**				.13
	Underweight	58	4 (6.9)	
	Normal weight	270	51 (18.9)	
	Overweight	82	12 (14.6)	
	Obese	46	6 (13.0)	
**Smoking**				.56
	Nonsmoker	420	65 (15.5)	
	Previous smoker	32	7 (21.9)	
	Current smoker	4	1 (25.0)	
**Physical activities**				.14
	Never	82	19 (23.2)	
	Sometimes	230	30 (13.0)	
	Usually	124	22 (17.7)	
	Every day	20	2 (10.0)	
**Inadequate dietary diversity (DDS^b^ score<7)**				.17
	No	374	64 (17.1)	
	Yes	82	9 (11.0)	
**Poor sleep quality (PSQI^c^ score>5)**				.003
	No	282	34 (12.1)	
	Yes	174	39 (22.4)	
**Time spent on reading COVID-19 news (hours)**				<.001
	<0.5	23	5 (21.7)	
	0.5-2	247	25 (10.1)	
	2-3	132	26 (19.7)	
	≥3	54	17 (31.5)	

^a^N/A: not applicable.

^b^DDS: dietary diversity score.

^c^PSQI: Pittsburgh Sleep Quality Index.

### Association Between Media Use, Lifestyles, and the Risk of Miscarriage

**Figure 2:**
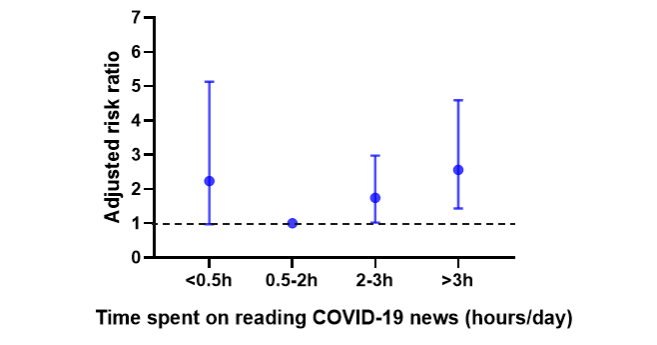
The adjusted risk ratios of association between media use about COVID-19 and the risk of miscarriage by a log-binomial regression model. The 0.5-2 hours group was the reference group.

We observed a U-shape relationship between media use about COVID-19 and the risk of miscarriage (see Figure 2). In the multivariable model, poor sleep quality (adjusted RR 2.06, 95% CI 1.24-3.44; *P*=.006), 2-3 hours of media use daily (adjusted RR 1.74, 95% CI 1.02-2.97; *P*=.04), and >3 hours of media use daily (adjusted RR 2.56, 95% CI 1.43-4.59; *P*=.002) were associated with miscarriage (see Table 3).

**Table 3:** Risk factors related with miscarriage by a log-binomial regression model.

Factors		Participants, n	Miscarriage, n (%)	Multivariable model^a^	
				Adjusted RR^b^ (95% CI)	*P* value
**Time spent on reading COVID-19 news (hours)**					
	<0.5	23	5 (21.7)	2.23 (0.97-5.13)	.06
	0.5-2	247	25 (10.1)	1 (reference)	N/A^c^
	2-3	132	26 (19.7)	1.74 (1.02-2.97)	.04
	>3	54	17 (31.5)	2.56 (1.43-4.59)	.002
**Poor sleep quality (PSQI^d^ score≥5)**					
	No	282	34 (12.1)	1 (reference)	N/A
	Yes	174	39 (22.4)	2.06 (1.24-3.44)	.006

^a^In the multivariable model, we adjusted sociodemographic characteristics (age, educational level, region, and family income), history of previous health (cesarean section, preterm birth, miscarriage, and fist pregnancy), prepregnancy BMI, smoking, physical activities, dietary diversity, and sleep quality. The covariates with *P*<.05 are shown in this table.

^b^RR: risk ratio.

^c^N/A: not applicable.

^d^PSQI: Pittsburgh Sleep Quality Index.

### Sensitivity and Subgroup Analyses

In the sensitivity analysis, the association between excessive media use about COVID-19 and the risk of miscarriage was stable (see Multimedia Appendix 1). In the subgroup analysis, the risk of miscarriage was significantly higher in the <0.5 hours media use group among women living in urban areas (*P*=.04), who had a history of miscarriage (*P*=.005), and who had advanced maternal age (*P*<.001), and was significantly higher in the >3 hours media use group among women living in rural areas (*P*=.001), who had no history of miscarriage (*P*<.001), and who were younger (*P*=.004; see Multimedia Appendix 2).

## Discussion

### Principal Findings

To our knowledge, this is the first study exploring the status of social media use and lifestyles among pregnant women during the COVID-19 pandemic and assessing their associations with the risk of miscarriage in a prospective cohort study. Our results showed a significant association between excessive media use, unhealthy lifestyle, and the risk of miscarriage in Chinese pregnant women. No previous study has assessed the status of social media use during the COVID-19 pandemic among pregnant women. There were some studies that examined the impact of exposure to COVID-19 information on the metal health status among the nonpregnant population (eg, internet users and factory workers) [18]. Nekliudov et al [15] conducted a cross-sectional online survey in a large Russian population using multiple social media platforms and found that time spent following news on COVID-19 was strongly associated with an increased anxiety. To be specific, compared to less than 30 minutes spent reading COVID-19 news per day, the 1-2 hours group was associated with a 5.46 (95% CI 5.03-5.90) point difference, the 2-3 hours group with a 7.06 (95% CI 6.37-7.74) point difference, and the >3 hours group with an 8.65 (95% CI 7.82-9.47) point difference [15]. Pan et al [19] did a cross-sectional web-based survey of 3035 factory workers at the beginning of work resumption following the COVID-19 outbreak in Shenzhen, China. They found that higher overall information exposure to COVID-19 was associated with higher depression symptoms. Similar with the previous studies, we found that, compared with the 0.5-2 hours media use group, the risk of miscarriage was significantly higher in the 2-3 hours media use group (adjusted RR 1.74) and >3 hours media use group (adjusted RR 2.56). One possible explanation of these findings was that women who spend too much time on social media might be more likely to have unhealthy lifestyles (eg, fewer physical activities, inadequate dietary diversity, and poor sleep quality), which might be related with miscarriage. Another possible explanation of these findings was that pregnant women with exposure to the excessive media information were more likely to have psychological problems (eg, depression and anxiety), which might also increase the risk of miscarriage. Berthelot et al [20] found that women in the COVID-19 pandemic were more likely to present clinically significant levels of depression and anxiety symptoms (odds ratio 1.94) than pre–COVID-19 women in Canada. The underlying mechanism on the relationship between excessive media information and the risk of miscarriage needs to be explored in the future.

It is worth noting that a U-shape relationship between time spent on reading COVID-19 news per day and the risk of miscarriage was found in our study. The risk of miscarriage was significantly higher in the <0.5 hours media use group among women who lived in urban areas, had a history of miscarriage, and had advanced maternal age, and significantly higher in the >3 hours media use group among women who lived in rural areas, had no history of miscarriage, and were younger. Previous history of miscarriage and advanced maternal age are both risk factors for miscarriage in the literature [12]. In a multicenter European study, the risk of miscarriage was found to be higher if the woman was 35 years or older, after adjustment for various factors (eg, reproductive history and country) [12]. Inadequate information on getting essential knowledge might be the explanation for the association between inadequate media use and the risk of miscarriage. In addition, this association was more obvious in populations with other risk factors of miscarriage. Our findings highlight the importance of obtaining moderate COVID-19–related information from social media and that either inadequate (<0.5 hours) or excessive (>3 hours) exposure to COVID-19–related information was not beneficial for the individuals.

In our study, we found that the mean time spent on reading COVID-19 news was 1.8 hours per day for the pregnant women, which was slightly shorter than the general population (2.4 hours) reported in other studies [21]. The worry of radiation from phones or computers among pregnant women might be related to their relatively shorter time spent on social media. Social media has an imperative role in the world that can provide a unified platform for public health communications, comprehensive health care education guidelines, and robust social distancing strategies while still maintaining social connections [21]. Meanwhile, fake news about COVID-19 on social networks could harm public health [22]. For individuals, it is difficult to effectively identify true and false information in the mass media. Just like a “double-edged sword,” social media needs to be used properly to help provide equal access to health care and end discrimination and social stigmatization. We also found that women with excessive media use were more likely to be previously pregnant, have no physical activity, have inadequate dietary diversity, and have poor sleep quality. Moreover, too much time spent on social media might also be related with unhealthy lifestyles, such as few physical activities, inadequate dietary diversity, and poor sleep. Keeping healthy lifestyles is helpful to prevent the occurrence of infectious disease and chronic disease. Our findings provide a clue for the early identification of a potentially high-risk population and miscarriage among pregnant women during the COVID-19 pandemic.

### Strengths and Limitations

The prospective cohort study design, controlling various risk factors related with miscarriage, and the first insight into the association of social media use with miscarriage are the strengths of this study. However, there are several limitations in this study. First, we did not collect information on the time spent on different kinds of social media (eg, official and unofficial web-based media, newspapers, and magazines) and individual behaviors on COVID-19 prevention. Second, genetic and psychological factors associated with miscarriage were not investigated in this study. The results need to be interpreted with caution. The potential intermediation role of psychological factors on the association between time spent on reading COVID-19 news and miscarriage needs to be explored in further studies. Third, this study was a single-center cohort conducted in China. A multicenter cohort study is needed to verify the findings in this study. Despite these limitations, our findings are helpful to better understand the role of social media and lifestyle on health among pregnant women during the COVID-19 pandemic. The role of media and public health communications needs to be correctly understood and explored further, as they will be an essential tool for delivering information and combating COVID-19 and health promotion, especially for vulnerable populations such as pregnant women.

### Conclusions

Pregnant women spent about 2 hours a day reading COVID-19 news in the early stage of the COVID-19 pandemic in China. Pregnant women with excessive media use were more likely to having no physical activity, inadequate dietary diversity, and poor sleep quality. Excessive media use and poor sleep quality were associated with a higher risk of miscarriage. Our findings highlight the importance of healthy lifestyles during the COVID-19 pandemic.

## Appendix

Multimedia Appendix 1 Supplemental Table: Sensitivity analysis on the association between media use about COVID-19 and the risk of miscarriage.

Multimedia Appendix 2 Supplemental Figure: Subgroup analysis of the association between media use about COVID-19 and miscarriage.

## Abbreviations

DDS: dietary diversity score
PSQI: Pittsburgh Sleep Quality Index
RR: risk ratio
