# Supplementary material for: Social Media Use, Unhealthy Lifestyles, and the Risk of Miscarriage Among Pregnant Women During the COVID-19 Pandemic: Prospective Observational Study
Source: JMIR Public Health Surveill. 2021 Jan 5;7(1):e25241. doi: 10.2196/25241 (PMC7787689; doi:10.2196/25241)
Supplement: Multimedia Appendix 1 [file publichealth_v7i1e25241_app1.docx]

**Supplemental table 2.** **Sensitivity analysis on the association between media use about COVID-19 and the risk of miscarriage.**

| Factors | N | Miscarriage (%) |  | Multivariable model | |
| --- | --- | --- | --- | --- | --- |
|  |  |  |  | Adjusted RR (95% CI) | *P* value |
| Time spent on reading COVID-19 news | | | | | |
| <0.5 hours | 23 | 5 (21.7) |  | 2.30 (0.97-5.45) | .059 |
| 0.5-2 hours | 247 | 25 (10.1) |  | 1 (reference) | - |
| 2-3 hours | 132 | 26 (19.7) |  | 1.87 (1.11-3.16) | .019 |
| ≥3 hours | 54 | 17 (31.5) |  | 2.82 (1.56-5.07) | .001 |
| Sleep quality |  |  |  | 1.12 (1.02-1.23) | .023 |

Abbreviations: RR, risk ratio; CI, confidence interval. In the multivariable model, we adjusted social-demographic characteristics (age, educational level, region, and family income), history of previous health (cesarean section, preterm birth, miscarriage, and fist pregnancy), pre-pregnancy BMI, smoking, physical activities, dietary diversity, and sleep quality (as a continuous variable).
