# Supplementary figures and images for: Social Media Use, Unhealthy Lifestyles, and the Risk of Miscarriage Among Pregnant Women During the COVID-19 Pandemic: Prospective Observational Study
Source: JMIR Public Health Surveill. 2021 Jan 5;7(1):e25241. doi: 10.2196/25241 (PMC7787689; doi:10.2196/25241)

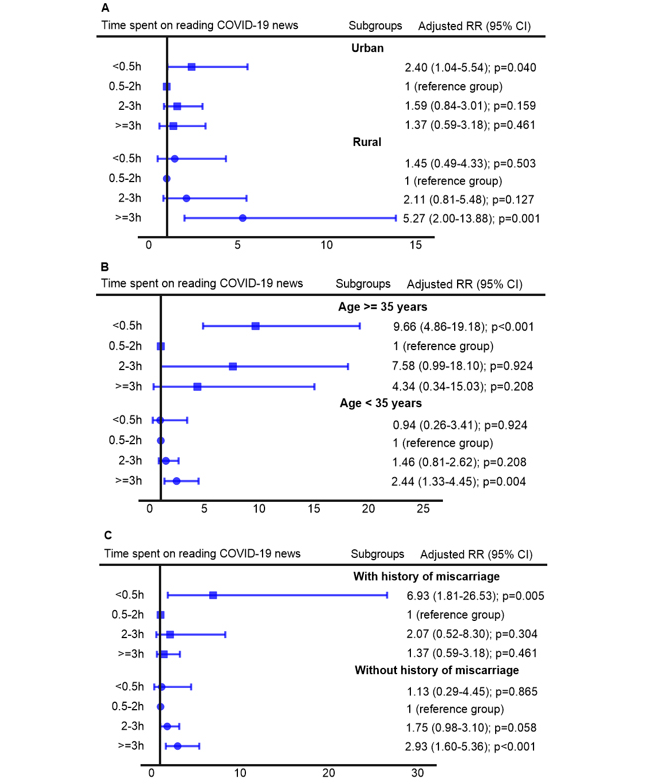

Supplement: Multimedia Appendix 2 [file publichealth_v7i1e25241_app2.png]
